# IL-10 Producing B Cells Ability to Induce Regulatory T Cells Is Maintained in Rheumatoid Arthritis

**DOI:** 10.3389/fimmu.2018.00961

**Published:** 2018-05-03

**Authors:** Julie Mielle, Rachel Audo, Michael Hahne, Laurence Macia, Bernard Combe, Jacques Morel, Claire Daien

**Affiliations:** ^1^Montpellier University, Montpellier, France; ^2^Institut de Génétique Moléculaire de Montpellier, University of Montpellier, CNRS, Montpellier, France; ^3^Department of Rheumatology, CHU de Montpellier, Montpellier, France; ^4^Charles Perkins Centre, Discipline of Pathology, School of Medical Sciences, The University of Sydney, Sydney, NSW, Australia

**Keywords:** regulatory B cell, autoimmunity, interleukin 10, programmed death-ligand 2, rheumatoid arthritis, B10

## Abstract

Despite growing evidence highlighting the relevance of increasing IL-10-producing B cells (B10^+^cells) in autoimmune diseases, their functions in patients are still unknown. The aim of this study was to evaluate the functions of CpG-induced B10^+^ cells isolated from healthy controls (HC) and rheumatoid arthritis (RA) patients, on naïve T cell differentiation. We demonstrated that CpG-induced B10^+^ cells from HC drove naïve T cell differentiation toward regulatory T cells (Treg cells) and IL-10-producing T cells (Tr1) through IL-10 secretion and cellular contacts. B10^+^ cells from HC did not decrease T helper 1 (Th1) nor and tumor necrosis factor α producing T cell (TNFα^+^ T cell) differentiation. We showed that in RA, B10^+^ cells could also induce Treg cells and Tr1 from naïve T cells. Contrary to HC, B10^+^ cells from RA patients increased naïve T cell conversion into Th1. Interestingly, PD-L2, a programmed death-1 (PD-1) ligand that inhibits PD-L1 and promotes Th1 differentiation, was overexpressed on RA B10^+^ cells compared to HC B10^+^ cells. Together, our findings showed that CpG-induced B10^+^ cells may be used to increase Treg cells in patients with RA. However, CpG may not be the most adequate stimuli as CpG-induced B10^+^ cells also increased inflammatory T cells in those patients.

## Introduction

Breakdown of immune tolerance can lead to the development of autoimmune diseases such as rheumatoid arthritis (RA). While the causes of such breakdown are not fully known, a key feature is the expansion of pro-inflammatory T cell subsets T helper 1 (Th1), Th17, and tumor necrosis factor α producing T cells (TNFα^+^ T cells) and, by contrast, the decrease of regulatory T (Treg) cell subsets. Both Tregs subsets, characterized either by the markers FoxP3 and CD4^+^CD25^hi^ CD127^low/neg^ (Treg cells) or by IL-10 secretion (Tr1), are able to limit the expansion of pro-inflammatory T cells. Similarly, auto-reactive B cells, which promote inflammation through the secretion of auto-antibodies, pro-inflammatory cytokines, or *via* antigen-presentation are increased, whereas regulatory B cells (Breg cells) are decreased. The role of Breg cells in tolerance has been established in both preclinical and clinical studies (1, 2). Indeed, the absence of Breg cells in mice has been shown to exacerbate the development of arthritis (3) while their adoptive transfer significantly decreases autoimmune disease severity in mouse models, such as experimental autoimmune encephalitis (4), colitis (5), and arthritis (6). Human studies have also showed impaired number and function of Breg cells in patients with auto immune and chronic inflammatory diseases (7–10). Thus, increasing the number of functional Breg cells in those patients could restore a balanced regulatory vs inflammatory response.

Different subsets of Breg cells can decrease inflammatory responses (4–6). In humans, immature transitional CD24^hi^CD38^hi^ B cells (7, 8, 11) and mature follicular CD24^hi^CD27^+^ B cells (12–14) were shown to decrease Th1, Th17, TNFα^+^ T cells and also to increase Treg cells and Tr1 through IL-10 production. However, the presence of CD24^hi^CD38^hi^ and CD24^hi^CD27^+^ B cells does not necessarily reflect their functionality. In fact, in patients with autoimmune diseases, while the abundance of CD24^hi^CD38^hi^ and CD24^hi^CD27^+^ B cells is comparable to those in healthy patients, they have lost the ability to induce Treg cells or to decrease Th1 and TNFα^+^ T cells (7, 8, 12). Thus, a marker for Breg cells which closely correlates with their functions is needed, both in healthy individuals and in patients. As both CD24^hi^CD38^hi^ and CD24^hi^CD27^+^ B cells are able to produce IL-10 after a stimulation with CpG, IL-10 production has been extensively used to define Breg cells, also known as B10^+^ cells (12, 15, 16). However, it is unknown whether any type of B cell secreting IL-10 has regulatory functions, in healthy subjects and in patients. Indeed, while the functions of CD24^hi^CD38^hi^ and CD24^hi^CD27^+^ B cells have been extensively described, CpG-induced B10^+^ cell regulatory functions remain fully elusive.

The objective of this study was to determine whether CpG-induced IL-10-producing B cells is a relevant functional definition for Breg cells in healthy subjects and in patients with RA.

## Materials and Methods

### Subjects

Healthy subjects were either blood donors or patients seen in the department of Rheumatology (Teaching hospital, Montpellier) for mild osteoarthritis or mechanical pain with no general pathology or infection and receiving no immunomodulatory drugs. To be included, patients with RA had to fulfill ACR/EULAR 2010 criteria, be free of biological disease-modifying anti-rheumatic drugs and have no glucocorticoid or less than 10 mg/day. All subjects signed a written informed consent for the study in accordance with the 2013 Declaration of Helsinki and as approved by the Medical Ethics Committee of Nimes hospital, France (CPP_2012-A00592-41). Characteristics of the controls and patients are detailed in Table 1.

**Table 1:** Characteristics of the subjects at inclusion.

	Healthy controls (*n* = 26)	RA patients (*n* = 31)
Age, median (IQR), years	44.0 (26.8, 52.5)	64.0 (52.0, 73.0)
Female, %	73.1	64.5
Disease duration, median (IQR), years	–	5.0 (1.3, 13.5)
Use of csDMARD (%)	–	62.1
Glucocorticoids, median (IQR), mg/day	–	5.0 (0.0, 10.0)
Presence of RF (%)	–	48.3
Presence of ACPA (%)	–	59.3

*IQR, interquartile ranges; csDMARD, classic disease-modifying anti-rheumatic drug; RF, rheumatoid factor; ACPA, anti-citrullinated protein antibody; RA, rheumatoid arthritis*.

### T Cell Isolation

Blood was collected in EDTA K2 tubes. PBMCs were isolated from 10 mL of whole blood using Ficoll-Paque Plus (GE Healthcare, Aulnay-sous-bois, France), cultivated on 96-well plate at 1.5 × 10^6^ cells/mL for 24 h in filtered complete media (RPMI 1640 with 10% fetal calf serum, penicillin and streptomycin). The day after, PBMCs were stained and sorted using FACS ARIA III (BD biosciences). To isolate CD4^+^CD25^−^ cells, we used FITC-conjugated anti-CD4 (clone RPA-T4, BD Pharmingen) and PE-conjugated anti-CD25 (clone BC96, ebioscience, Thermofisher, USA) antibodies. To sort naïve CD4^+^CD45RA^+^CD62L^+^ cells, we used APC eFluor 780-conjugated anti-CD4 (RPA-T4, ebioscience), FITC-conjugated anti-CD62L (clone DREG-56, BD Pharmingen), and PerCP Cy5.5-conjugated anti-CD45RA (clone HI100 BD Pharmingen) antibodies. Dead cells were excluded using DAPI staining. Gating strategies are shown Figure S1 in Supplementary Material.

### B10^+^ Cell Isolation

B cells were isolated from 50 mL of whole blood using RosetteSep human B enrichment (StemCell, Grenoble France), followed by a Ficoll separation. As tested previously (12), B cells were cultivated in complete media at 3 × 10^6^ cells/mL and stimulated with plate bound CD40L (1 µg/mL, Peprotech, Neuilly sur Seine, France), and CpG (10 µg/mL, ODN 2006, InvivoGen, Toulouse, France) for 24 h to generate B10^+^ cells. IL-10 secretion was activated by adding Phorbol 12-myristate 13-acetate (PMA) (0.1 µg/mL, BioVision, Nanterre, France) and ionomycin (0.5 µg/mL, BioVision) for the last 4 h of culture. After stimulation, B10^+^ cells were stained using IL-10 secretion assay from Miltenyi biotec (Paris, France) according to the manufacturer’s instructions. Briefly, B cells were first incubated for 5 min on ice with IL-10-specific catch reagent that can attach both leukocyte surface and IL-10. Then B cells were incubated during 45 min at 37°C, to allow IL-10 secretion. The secreted IL-10 bound to the IL-10 Catch Reagent attached on secreting cells. IL-10 secreting cells were then detected using a second IL-10-specific antibody, the IL-10 Detection Antibody conjugated to R-phycoerythrin (PE). At this last step, an APC-conjugated anti-CD19 antibody (clone HIB19, BD Pharmingen) was also added. After staining, B cells were sorted using FACS ARIA III (BD bioscience), into two populations: B cells with positive IL-10 staining (referred to as B10^+^ cells) and cells with negative IL-10 staining (referred to as B10^neg^cells). Dead cells were excluded using DAPI staining. Gating strategies are shown in Figure S1 in Supplementary Material.

### Cytokines Assessment

PBMCs from healthy individuals were stimulated by CpG (10 µg/mL) for 24 h. PMA and ionomycin were then added for the last 4 h of culture and BFA (10 µg/mL, Sigma Aldrich) was added for the last 2 h. PBMCs were then stained using FITC or V450-conjugated anti CD19 (clone HIB19, BD Pharmingen) and Fixable Viability Dye eFluor 506 (Thermofisher) before fixation (cytofix/cytoperm buffer BD biosciences) and permeabilization (Perm wash buffer, BD Pharmingen). Intracellular staining for IL-10, IFNγ, and TNFα was then performed using APC-conjugated anti IL-10 (clone JES3-19F1, BD Pharmingen) and PeCy7-conjugated anti-IFNγ PeCy7 (4SB3, BD Pharmingen) or anti-TNFα (clone MAb11, BD Pharmingen). Percentages of IFNγ and TNFα positive cells among B10^+^ and B10^neg^ cells were measured by flow cytometry, using FACS CANTO II (BD biosciences). Gating strategy and representative flow cytometry dot plot are shown in Figure S2 in Supplementary Material. We also measured cytokines concentrations in supernatants from isolated B10^+^ and B10^neg^ cells. To do this, B10^+^ and B10^neg^ cells were sorted as described above, and kept in culture for 24 h. Then, cytokine dosages in supernatants were performed using Human IFN-γ ELISA MAX Deluxe and Human TNF-α ELISA MAX Deluxe (BioLegend, San Diego, CA, USA) according to the manufacturer’s instructions.

### Cell Co-Culture and Conversion Assay From Naïve T Cells to Treg, Tr1, Th1, and TNFα^+^ T Cells

Isolated T cells were cultured either alone or in a ratio 1:1 with autologous sorted B cell subsets (B10^+^ or with B10^neg^ cells) in complete media, stimulated with plate bound anti-CD3 (dose 1 µg/mL) during 72 h. For Treg cell staining, FITC or APC-efluor780-conjugated anti-CD4, PE-conjugated anti-CD25 and PeCy5-conjugated anti-CD127 (clone eBioRDR5, eBioscience) antibodies were used. Dead cells were excluded using DAPI staining. Treg cells were defined as CD4^+^CD25^+^CD127^low/neg^.

Tr1, Th1, and TNFα^+^ T cell intracellular staining was performed after activation with PMA and ionomycin the last 4 h of culture and then BFA was added for the last 2 h of culture. Cells were stained with FITC conjugated anti-CD4 (clone RPA-T4, BD Pharmingen) or APC-efluor780 conjugated anti-CD4 (clone RPA-T4 ebioscience) antibodies before fixation and permeabilization. T cells cytokine secretion was then assessed using APC-conjugated anti-IFNγ (clone B27, BD Pharmingen), PeCy7-conjugated anti-TNFα (clone MAb11, BD Pharmingen), and PE-conjugated anti-IL-10 (clone JES3-9D7, eBioscience) antibodies. A fixable viability dye efluor506 discriminant was used according to the manufacturer’s instructions. Data were acquired by a FACS CANTO II. Th1 were defined as CD4^+^IFNγ^+^ cells, TNFα^+^ T cells were defined as CD4^+^TNFα^+^ cells and Tr1 as CD4^+^IL-10^+^ cells.

Gating strategies for flow cytometry analysis were performed using FlowJo software V10 (Treestar, Ashland, OR, USA). First, PBMCs were gated based on forward scatter and side scatter, followed by exclusion of doublets and dead cells. Gating strategies are shown in Figure S1A in Supplementary Material.

### Contact Inhibition

B10^+^ cells and B10^neg^ cells were cultured with CD4^+^CD25^−^ cells in 24-well plate either together or separated with a 0.4-µm Pore Polycarbonate Membrane Insert (Corning Inc., New York, NY, USA) in 600 µL of complete media. Treg cells were then assessed by flow cytometry after 72 h.

### Blocking Antibodies

B cells were first incubated with Blocking antibody against PDL-1 (CD274, clone MIH1, BD Pharmingen) or PDL-2 (CD273, R&D Systems, Lille, France) before the co-culture with T cells. Anti-IL-10 (clone JES3-9D7, BD Pharmingen), anti CD80 (B7.1, clone 2D10.4, ebioscience), and anti CD86 (clone IT2.2, BD Pharmingen) were added directly into the media. All the inhibitors were used at 10 µg/mL during 72 h. Percentages of Treg cells were assessed by flow cytometry after 72 h.

### Statistical Analysis

Comparison between paired data were performed using Wilcoxon matched pairs test. Significance was ascribed to *p* values <0.05. To compare variations between healthy controls (HC) and patients, we expressed data as median ± interquartile range (IQR) 25–75 and significance was assessed using Mann–Whitney test. All analyses were performed in Graph Pad Prism 5 (San Diego, CA, USA).

## Results

### CpG-Induced B10^+^ Cells Produced More Pro-Inflammatory Cytokines Than B10^neg^ Cells in HC

**Figure 1:**
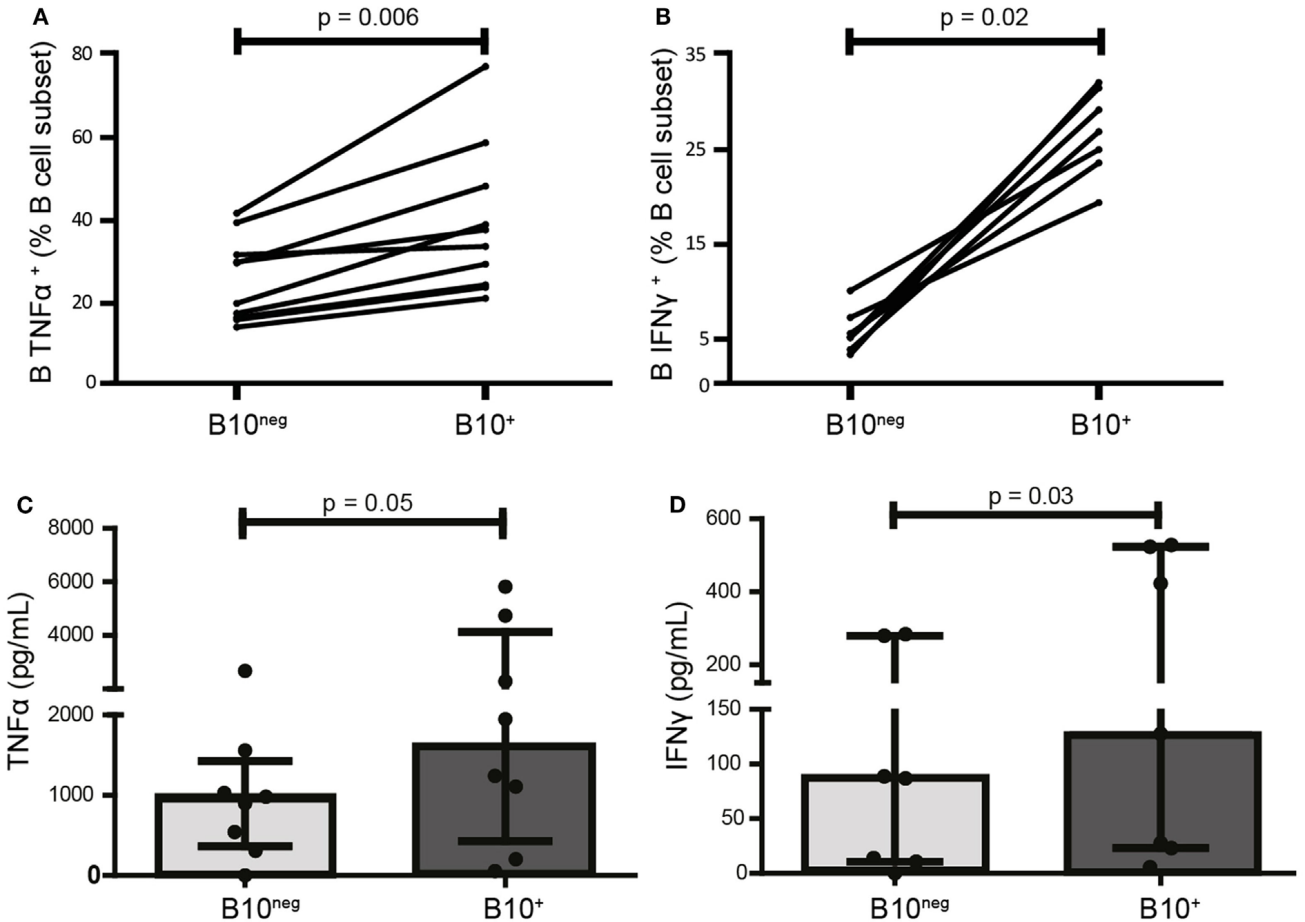
CpG-induced B10^+^ cells are more prone to produce pro-inflammatory cytokines than B10^neg^ cells. PBMCs were isolated from whole blood of healthy donors, stimulated 24 h with CpG, and stained intracellularly for tumor necrosis factor α (TNFα) **(A)** IFNγ **(B)**, and IL-10. Cytokine production was assessed by flow cytometry in B10^+^ subset and B10^neg^ subset. In **(C,D)**, TNFα and IFNγ concentration in the supernatants after 24 h of culture of isolated B10^+^ and B10^neg^ were measured by ELISA. Results are shown as median ± interquartile ranges and Wilcoxon matched pairs test was used for statistical analysis. *n* = 7–11.

TLR9 ligation by CpG is the most potent and the most commonly used inducer of B10^+^ cells. However, it also promotes release of pro-inflammatory cytokines by B cells (17). As the effect of CpG on the release of pro-inflammatory cytokines by Breg cells is unknown, we first studied the secretion profile for TNFα and IFNγ of B10^+^, induced by CpG, isolated from HC. Despite their secretion of the anti-inflammatory cytokine IL-10, B10^+^ are also significantly more TNFα^+^ and IFNγ^+^ than B10^neg^ (TNFα^+^ median [IQR]: 35.80% [24.35; 50.93] vs 24.90% [16.48; 33.73]; *p* = 0.006; *n* = 10 and IFNγ^+^ median: 26.90% [23.60; 31.50] vs 5.20% [3.89; 7.27]; *p* = 0.02; *n* = 7) (Figures 1A,B; Figure S2 in Supplementary Material). These results were then confirmed by ELISA. Indeed, B10^+^ secreted more TNFα (1,595 pg/mL [429; 4,131] vs 942 pg/mL [367; 1,426]) and IFNγ (128 pg/mL [29; 524] vs 87 pg/mL [11; 280]) than B10^neg^ cells (Figures 1C,D).

### CpG-Induced B10^+^ Cells Could Convert Naïve T Cells Into Tregs in HC

**Figure 2:**
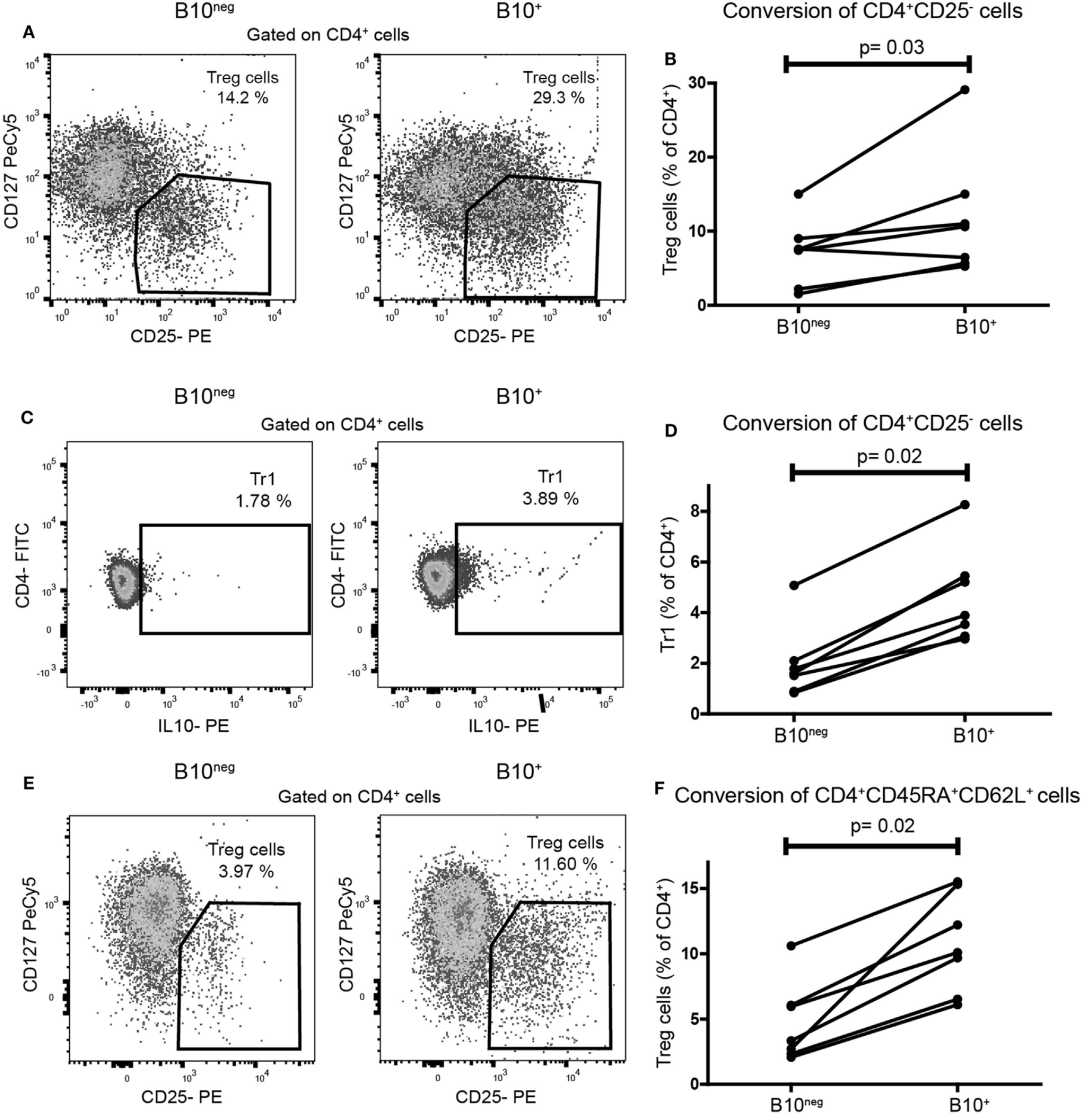
CpG induced B10^+^ cells from healthy subjects increased regulatory T (Treg) cells and Tr1 differentiation from naïve T cells. Sorted B10^+^ cells or B10^neg^ cells were co-cultured for 72 h with autologous CD4^+^CD25^−^cells or CD4^+^CD45RA^+^CD62L^+^ naïve T cells stimulated with anti CD3. Treg cells, defined as CD4^+^CD25^hi^CD127^lo/neg^, and Tr1, defined by CD4^+^IL-10^+^, were assessed by flow cytometry. Representative flow cytometry plot (gated on CD4^+^ cells) and percentages of CD4^+^CD25^−^ cells conversion by B10^+^ or B10^neg^ into Treg cells **(A,B)** or Tr1 **(C,D)** cells are presented. **(E,F)** Flow cytometry plot and graph presenting the conversion of CD4^+^CD45RA^+^CD62L^+^ by B10^+^ cells or B10^neg^ cells. Each connected pair of dots represents individual donor and results were obtained from independent experiments done in seven patients. *p* Values were calculated with Wilcoxon matched pairs test.

To assess the functional ability of CpG-induced B10^+^ cells to convert naïve T cells into Treg cells, we co-cultured sorted B10^+^ and B10^neg^ cells with purified autologous CD4^+^CD25^−^ T cells (gating strategies for sorting in Figure S1 in Supplementary Material). Proportion of Treg cells were assessed after 72 h by flow cytometry. We found that B10^+^ cells converted more CD4^+^CD25^−^ cells into Treg cells than B10^neg^ cells (median [IQR]: 10.6% [5.65; 15.00] vs 7.55% [2.18; 9.02] for B10^+^ and B10^neg^ cells, respectively; *p* = 0.03; *n* = 7) (Figures 2A,B). Similarly, B10^+^ cells could convert more CD4^+^CD25^−^ into Tr1, than B10^neg^ cells (median [IQR]: 2.68% [1.19; 3.81] vs 1.69% [0.22; 2.39] for B10^+^ and B10^neg^ cells, respectively; *p* = 0.02; *n* = 7) (Figures 2C,D).

These results were confirmed in a co-culture using CD4^+^CD45RA^+^CD62L^+^ T cells, considered as a more accurate population of naïve T cells. Indeed, B10^+^ cells significantly increased the differentiation of naïve CD4^+^CD45RA^+^CD62L^+^ T cells into Treg cells, compared to B10^neg^ cells (10.10% [6.52; 15.30] vs 3.33% [2.33; 6.06] for B10^+^ and B10^neg^, respectively; *p* = 0.02; *n* = 7) (Figures 2E,F).

### IL-10 Is Necessary, but Not Sufficient, for CpG-Induced B10^+^ Cell Regulatory Functions

**Figure 3:**
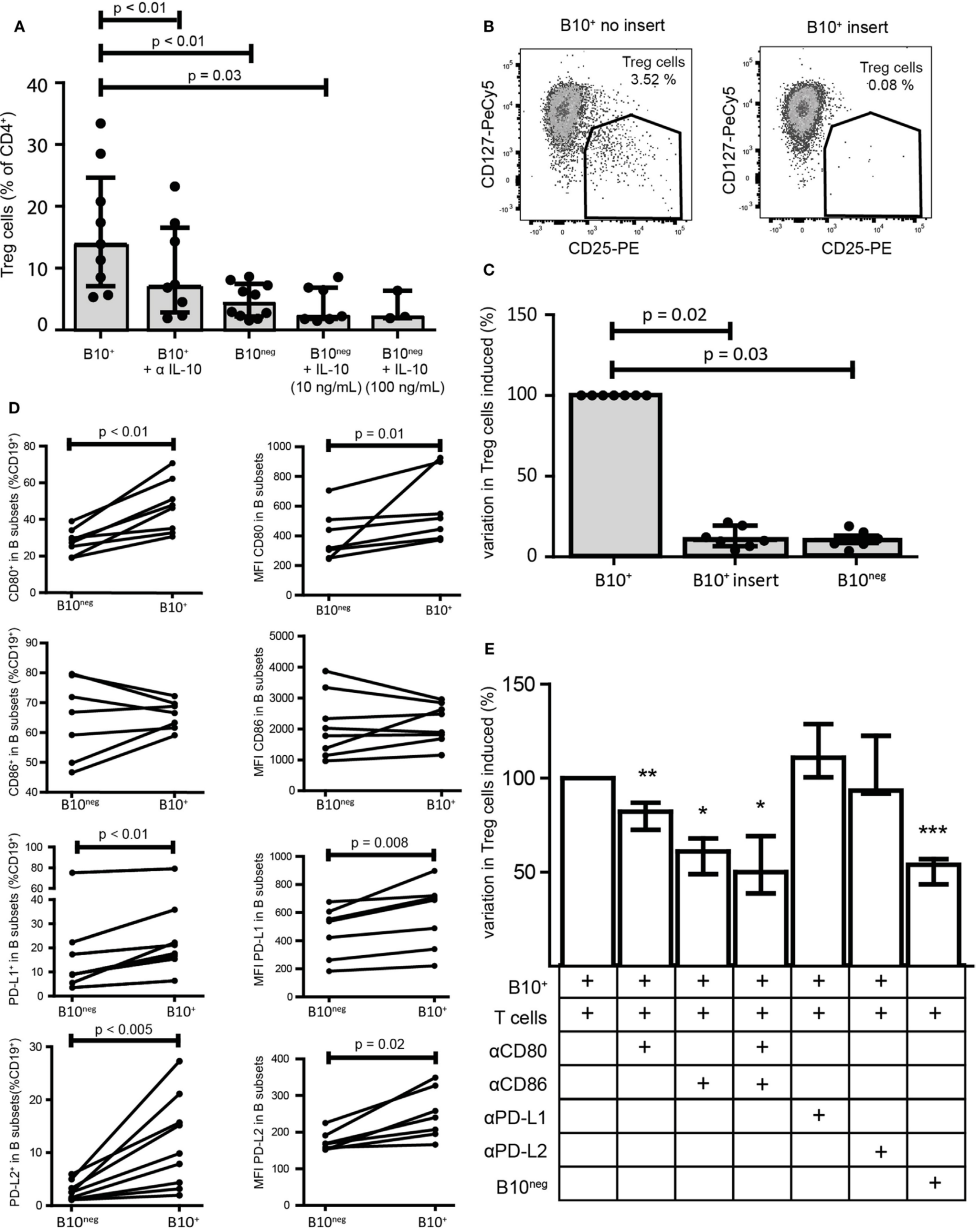
Regulatory functions of B10^+^ cells are mediated by IL-10 but also by cellular contacts. Sorted B10^+^ cells or B10^neg^ cells were co-cultured with autologous CD4^+^CD25^−^ cells for 72 h and regulatory T (Treg) cells (defined as CD4^+^CD25^hi^CD127^lo/neg^) proportion was assessed by flow cytometry. **(A)** Anti IL-10 and recombinant IL-10 at 10 or 100 ng/mL were added to the co-culture, *n* = 3–7. **(B,C)** Same co-cultures as **(A)** but contact between B10^+^ cells and CD4^+^CD25^−^ was prevented by membrane insert. **(D)** PBMCs were stimulated with CpG for 24 h and then CD80, CD86, PD-L1, and PD-L2 expression was assessed in B10^+^ and B10^neg^ subsets, *n* = 7–11. Percentages of B10^+^ and B10^neg^ cells positive for CD80, CD86, PD-L1, and PD-L2 are presented (left panel) and also median of fluorescence intensity (MFI) of CD80, CD86, PD-L1, and PD-L2 (right panel) in each subset. **(E)** Anti-CD80 (αCD80), anti-CD86 (αCD86), anti-PD-L1 (αPD-L1), and anti-PD-L2 (αPD-L2) were first incubated with B cells before co-culture. Variation in Treg cells, induced by each inhibitor, was expressed as a percentage, compared to the co-cultured B10^+^/CD4^+^CD25^−^ cells. Cumulative results for seven donors are shown for each condition, Wilcoxon matched pairs test. *p < 0.05, **p < 0.01, ***p < 0.0005.

It has been shown that CD24^hi^CD38^hi^ and CD24^hi^CD27^+^ B cell regulatory functions were mainly mediated by IL-10 and cell contacts. To assess the respective effect of IL-10 and cellular contacts in our system, we first neutralized IL-10 with a blocking antibody. IL-10 blocking significantly reduced CD4^+^CD25^−^ conversion into Treg cells (7.09% [2.86; 16.55] with anti-IL-10 antibody vs 13.90% [7.09; 24.65] without it; *p* = 0.008; *n* = 8) (Figure 3A). However, Treg cell conversion was not solely attributable to IL-10 since addition of recombinant IL-10 (10 and 100 ng/mL) to B10^neg^ cells could not lead to the same increase in Treg cells (Figure 3A). To test whether cellular contacts were needed to generate Treg cells, we separated B10^+^ and T cells with a membrane insert, so that cytokine transfers were still effective but direct contacts were prevented. We found that cellular contacts were essential since adding insert to the co-cultures lead to a drastic decrease in Treg cells (Treg decrease of 90% [80.56; 93.33]; *p* = 0.02; *n* = 7) (Figures 3B,C).

To further characterize the cellular interactions between B10^+^ cells and T cells, we assessed the surface expression on B10^+^ cells and on B10^neg^ cells of the co-stimulatory molecules CD80, CD86, and the program death molecule ligands 1 and 2 (PD-L1 and PD-L2), known to trigger Treg cell functions. We found that all of them, with the exception of CD86, were more often expressed and with a higher level of expression on B10^+^ cells compared to B10^neg^ cells (Figure 3D; Figure S3 in Supplementary Material). We then assessed the effect of blocking these co-stimulatory molecules on Treg cell differentiation. We found a decrease in Treg cells when blocking CD86 (−38.94% [32.05; 51.11]; *p* = 0.03; *n* = 7), CD80 (−17.84% [13.02; 27.40]; *p* = 0.005; *n* = 11), and both (−49.90% [30.78; 61.33]; *p* = 0.03; *n* = 6). However, blocking either PD-L1 or PD-L2 did not alter B10^+^ cell ability to increase Treg cells (+11.00% [−0.40; +28.80] with anti-PD-L1; *p* = 0.25, *n* = 4; −6.52% [−22.60; 8.20] with anti-PD-L2; *p* = 1.00; *n* = 3) (Figure 3E) suggesting that co-stimulatory molecules rather than programmed death-1 (PD-1) ligands are critical for CpG-induced B10^+^ cell induction of Treg cells.

### CpG-Induced B10^+^ Cells Did Not Suppress Th1 Nor TNFα-Producing T Cells in HC

**Figure 4:**
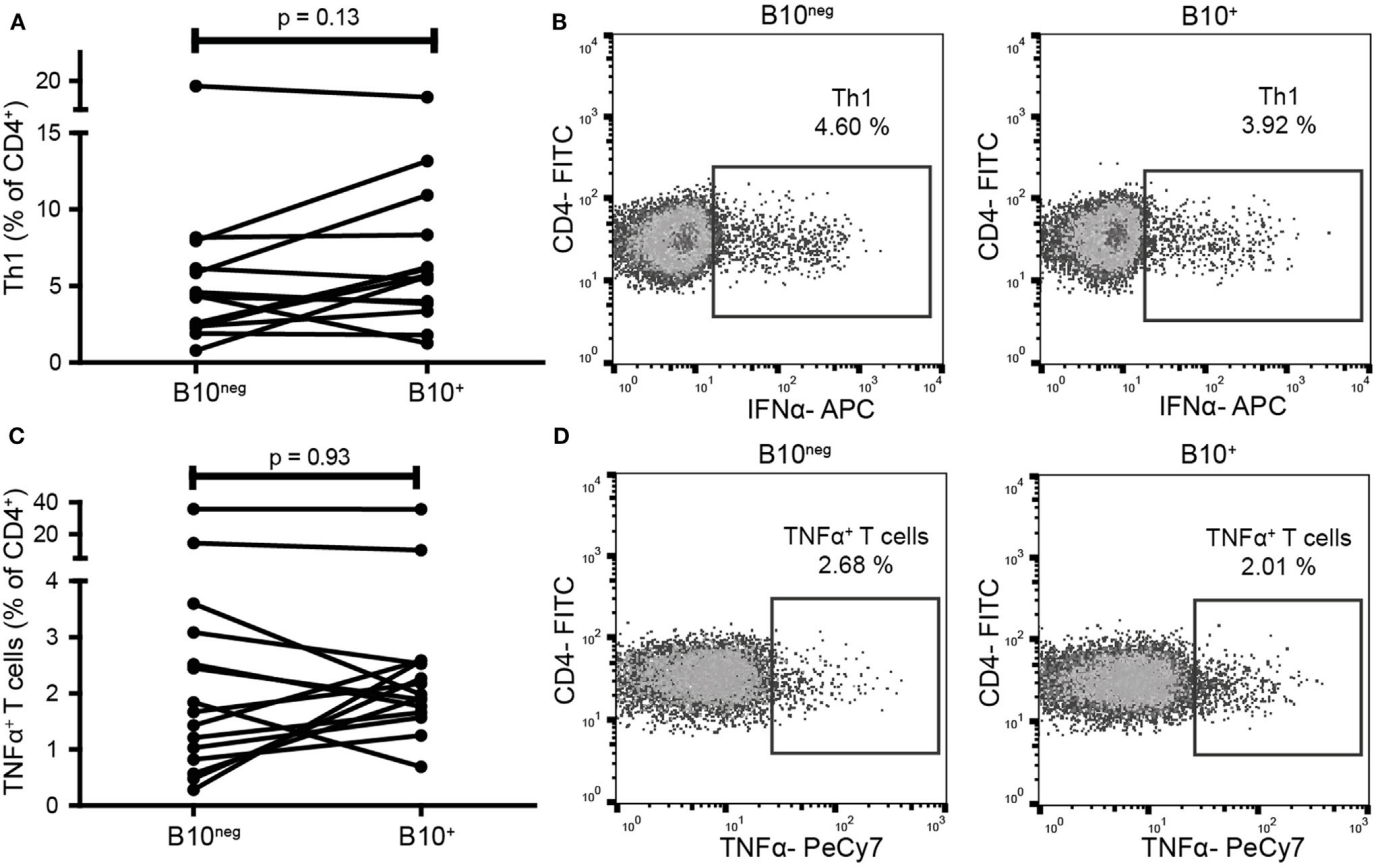
B10^+^ cells did not decrease T helper 1 (Th1) nor TNFα^+^ T cells frequencies. CD4^+^CD25^−^ cells were sorted and cultured in presence of B10^+^ or B10^neg^ cells. Th1 **(A,B)** and TNFα^+^ T cells **(C,D)** percentages were assessed after 72 h. Wilcoxon matched pairs test. *N* = 8.

As CD24^hi^CD38^hi^ and CD24^hi^CD27^+^ B cells can decrease pro-inflammatory Th1 and TNFα^+^ T cells, we assessed if B10^+^ cells had the same function. We thus co-cultured B10^+^ and B10^neg^ cells with CD4^+^CD25^−^ cells. In B10^+^ cell co-cultures, we did not observe any significant decrease in Th1 (defined as CD4^+^IFNγ^+^) frequency (5.645% [3.73; 8.98]) compared to B10^neg^ cell co-cultures (4.32% [2.36; 6.60]; *p* = 0.14; *n* = 14) (Figures 4A,B) nor in TNFα^+^ T cells frequency (1.98% [1.66; 2.58] vs 1.67% [0.82; 3.09] with B10^+^ and B10^neg^ cells, respectively; *p* = 0.93; *n* = 15) (Figures 4C,D).

### B10^+^ Cells From RA Patients Effectively Convert Naïve T Cells Into Treg Cells and Tr1 but Also Increased the Proportion of Th1

**Figure 5:**
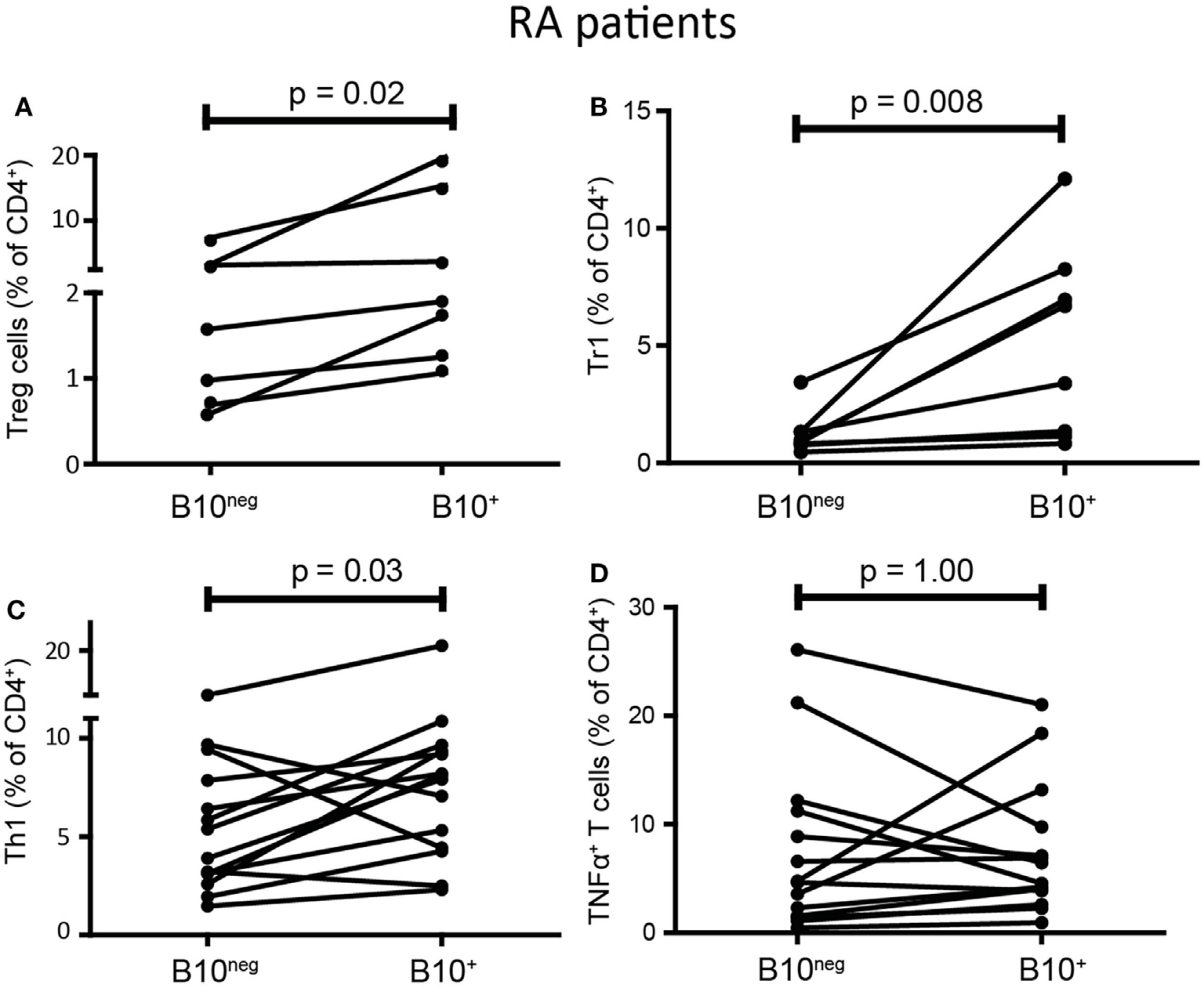
B10^+^ cells from rheumatoid arthritis (RA) patients could induce regulatory T (Treg) cells and Tr1 but also increased T helper 1 (Th1). CD4^+^CD25^−^ T cells were cultured with autologous B10^+^ or B10^neg^ cells from RA patients and percentages of Treg cells **(A)**, Tr1 **(B)**, Th1 **(C)**, TNFα^+^ T cells **(D)** were assessed after 72 h. Wilcoxon matched pairs test. *N* = 7–8.

B10^+^ cell deficiency seems to play an important role in autoimmune diseases, however, B10^+^ functions in patients have poorly been studied. As observed in HC, we found that B10^+^ cells from RA patients induced more Treg cells than B10^neg^ cells (1.9% [1.27; 14.90] with B10^+^ vs 1.58% [0.72; 6.96] with B10^neg^ cells; *p* = 0.02; *n* = 7) (Figure 5A). RA B10^+^ cells induced also more Tr1 (5.04% [1.20; 7.94] with B10^+^ vs 0.93% [0.79; 1.33] with B10^neg^ cells; *p* = 0.008; *n* = 8) (Figure 5B). Surprisingly, B10^+^ cells from RA patients also induced more Th1 than B10^neg^ cells (8.03% [4.40; 9.42] with B10^+^cells vs 4.65% [3.00; 8.25] with B10^neg^ cells; *p* = 0.033; *n* = 14) (Figure 5C). However, like in HC, RA B10^+^ cells have no effect on TNFα^+^ T cell differentiation (5.52% [3.56; 10.61] with B10^+^ cells vs 4.70% [1.54; 11.47] with B10^neg^ cells; *p* = 1; *n* = 14) (Figure 5D). Taken together, these results suggest that IL-10 secretion is a consistent marker, even in patients, of Breg cell functions on Treg cells and Tr1, but not on Th1.

### B10^+^ Cells From RA Patients Expressed a Higher Ratio of PD-L2 to PD-L1 Than in HC

**Figure 6:**
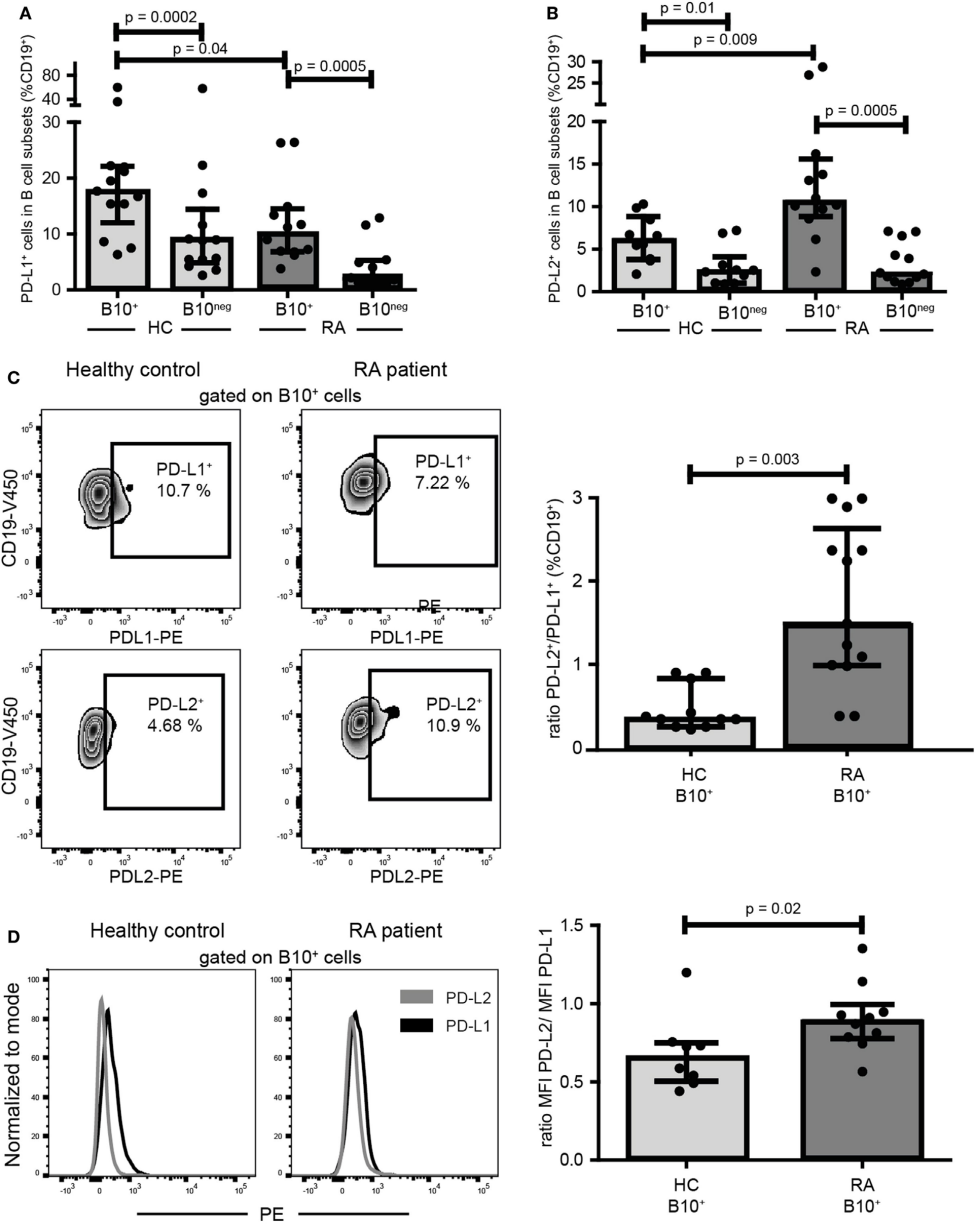
B10^+^ cells from rheumatoid arthritis (RA) patients expressed less PD-L1 and more PD-L2 than B10^+^cells of healthy controls (HC). PBMCs were cultured for 24 h with CpG. Percentages of PD-L1 **(A)** and PD-L2 **(B)** positive cells among B10^+^ and B10^neg^ cells subsets were assessed in HC and in RA patients by flow cytometry. Representative flow cytometry plots and ratios of B10^+^ PD-L2^+^/B10^+^PD-L1^+^ cells are shown in **(C)**. **(D)** Representative overlays and median of fluorescence intensity (MFI) showing PD-L2 and PD-L1 expression levels on B10^+^ cells. Medians and interquartile ranges are represented in the graphs. *p* values were calculated with Wilcoxon matched test for paired data or Mann–Whitney test for comparison between RA and HC.

PD-L1 was shown to inhibit Th1 differentiation whereas PD-L2 could promote it (18, 19). As RA B10^+^ cells induced Th1 differentiation while HC B10^+^ cells did not, we compared the expression of PD-L1 and PD-L2 on B10^+^ cells from RA and HC. Interestingly, RA B10^+^ cells were less PD-L1^+^ (10.10% [6.84; 14.53] vs 17.70% [12.02; 22.10]; *p* = 0.04; *n* = 12–13) and more PD-L2^+^ (10.60% [8.85; 15.60] vs 6.09% [3.81; 8.83]; *p* = 0.01; *n* = 10–12) (Figures 6A,B) leading to a higher ratio of PD-L2^+^ to PD-L1^+^ on RA B10^+^ cells (Figure 6C). This variation in PD-L1 and PD-L2 expression was also true when analyzing their expression levels by median of fluorescence intensity (Figure 6D). This might explain why RA B10^+^ cells induced Th1 differentiation but not HC B10^+^ cells.

## Discussion

The aim of the present study was to determine whether CpG-induced IL-10-producing B cells were a relevant functional definition for regulatory B cells in healthy subjects and in patients with RA. Functions of human B10^+^ cells have poorly been assessed in controls and in RA patients. We found that in HC, CpG-induced B10^+^ cells could increase the differentiation of naïve T cells into Treg cells and Tr1, through IL-10 secretion and cellular contacts. Using blocking antibodies, we showed that CD80 and CD86, but not the PD-1 ligands were required for B10^+^ cells to induce Treg cells. Interestingly, B10^+^ cells from RA patients could also increase the conversion of naïve T cells into Treg cells and Tr1. Thus, we showed that, unlike the CD24^hi^CD27^+^ or CD24^hi^CD38^hi^ B cells (8, 12), B10^+^ cells seems to be a consistent functional definition for B cells inducing Treg subsets.

Another important finding of this paper is that CpG-induced B10^+^ cells failed to limit Th1 and TNFα^+^ T cells differentiation, in contrast to previously described CD24^hi^CD27^+^ or CD24^hi^CD38^hi^ B cell functions (7, 8, 11, 12, 14). Most of the functional studies about CD24^hi^CD27^+^ or CD24^hi^CD38^hi^ B cells did not use any stimulation. CpG stimulation enhanced the production of IFNγ and TNFα by B cells which may counterbalance the effect of IL-10 on pro-inflammatory T cell differentiation. Therefore, we believe that CpG stimulation influenced the effect of B10^+^ cells on pro-inflammatory T cell differentiation. Along with this, Iwata et al. did not find any specific effect of CpG stimulated CD24^hi^CD27^+^ B cells on TNFα producing T cells differentiation (20). While type of stimulation is highly important, duration of stimulation may also modify B10^+^ function. Besides, Iwata described two types of B10^+^ cells; B cells that express IL-10 after only 5 h of stimulation, called B10 cells, and B cells that require 48 h of stimulation, called progenitor B10 cells. It is likely that, depending on the duration of the stimulation, different subtypes of B cells are expressing IL-10, and that these may have different functions. In our system, we did a 24 h stimulation, to minimize mortality (12). Thus, determining the best stimulation capable of inducing regulatory but not pro-inflammatory subsets should be address in further studies to optimize therapeutic potential of B10^+^ cells.

While B10^+^ cells of patients have a similar effect on Treg cell differentiation as in HC, RA B10^+^ cells increased Th1 cell frequency. A higher PD-L2 to PD-L1 ratio on RA B10^+^ cells compared to HC B10^+^ cells might explain this difference. In fact, Riccomi and Palma (19) showed that in co-culture of unexperienced B cells and CD4^+^ T cells, blocking PD-L2 inhibited IFNγ production whereas neutralizing PD-L1 enhanced proliferation and IFNγ production. In the same way, Karunarathne et al. (18) found that PD-L2 expressed on dendritic cells, by inhibiting PD-1 binding to PD-L1, increased T cell activation and improved Th1 response. In our study, because of the low number of B10^+^ cells in RA patients, we could not perform functional experiment to confirm PDLs involvement in Th1 differentiation.

Notably, PD-L1 and PD-1 blocking antibody have been used in cancer therapies (21). As PDLs seem to be implicated in Breg cell functions, further studies could be useful to support the choice between PD-L1 or PD-1 blocking antibodies in treating cancer.

Together, our findings show that CpG-induced B10^+^ cells may be used to increase Tregs in patients with RA but not to decrease pro-inflammatory T cells.

## Ethics Statement

This study was carried out in accordance with the recommendations of the local Ethic Committee. The protocol was approved by the Medical Ethics Committee of Nimes hospital, France (CPP_2012-A00592-41). All subjects gave written informed consent in accordance with the Declaration of Helsinki.

## Author Contributions

JMi designed and performed the experiments; collected, analyzed, and interpreted the data; and wrote the manuscript. RA designed and performed the experiments, interpreted the data. MH and LM critically read the manuscript and gave scientific advice. BC and JMo contributed to patient enrollment into the study and critically read the manuscript. CD obtained financial support, designed the experiments, conducted patient enrollment into the study, interpreted the data, and wrote the manuscript.

## Conflict of Interest Statement

The authors declare that the research was conducted in the absence of any commercial or financial relationships that could be construed as a potential conflict of interest.
