# Supplementary material for: IL-10 Producing B Cells Ability to Induce Regulatory T Cells Is Maintained in Rheumatoid Arthritis
Source: Front Immunol. 2018 May 3;9:961. doi: 10.3389/fimmu.2018.00961 (PMC5943500; doi:10.3389/fimmu.2018.00961)
Supplement: Supplementary file 1 [file presentation_1.PDF]

Supplementary Material

IL-10 producing B cells ability to induce regulatory T cells is maintained in rheumatoid arthritis

Julie Mielle[1,2,3,] Rachel Audo[1,2,3,] Michael Hahne[1,2], Laurence Macia[4], Bernard Combe[1,2,3], Jacques Morel[1,2,3], Claire Daien[1,2,3*]

*** Correspondence:** Dr. Claire Daien: c-daien@chu-montpellier.fr

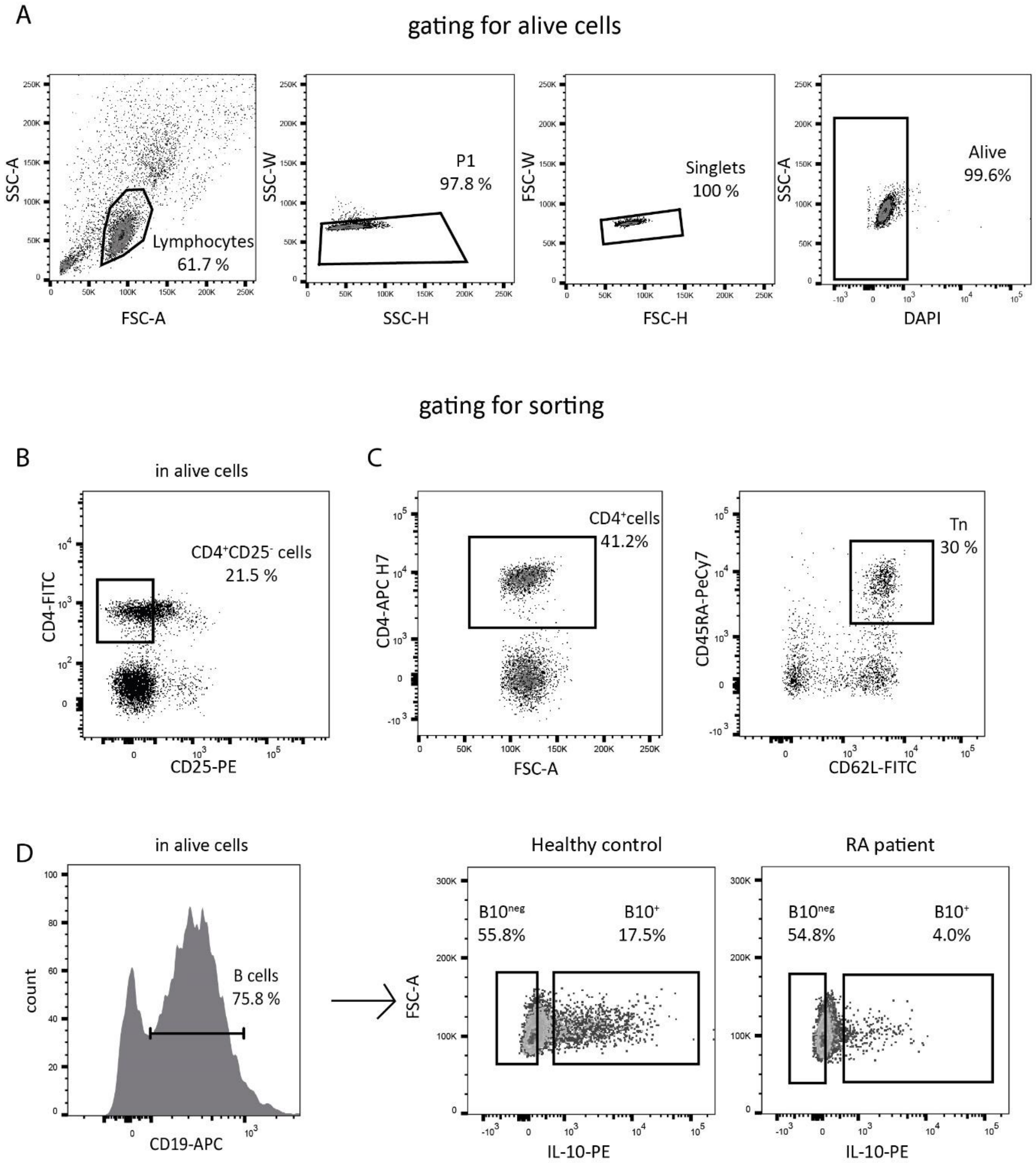

Supplementary Figure 1. Gating Strategy for flow cytometry. Example of gating strategy for singlets, alive cells **(A)**. Gating strategy for flow cytometry sorting of $CD4^+CD25^-$ T **(B)** or $CD4^+CD45RA^+CD62L^+$ naïve T cells **(C),** or $B10^+$ and $B10^{neg}$ cells **(D).** In **(D)** representative dot for healthy control (left) and RA patient (right) are presented.

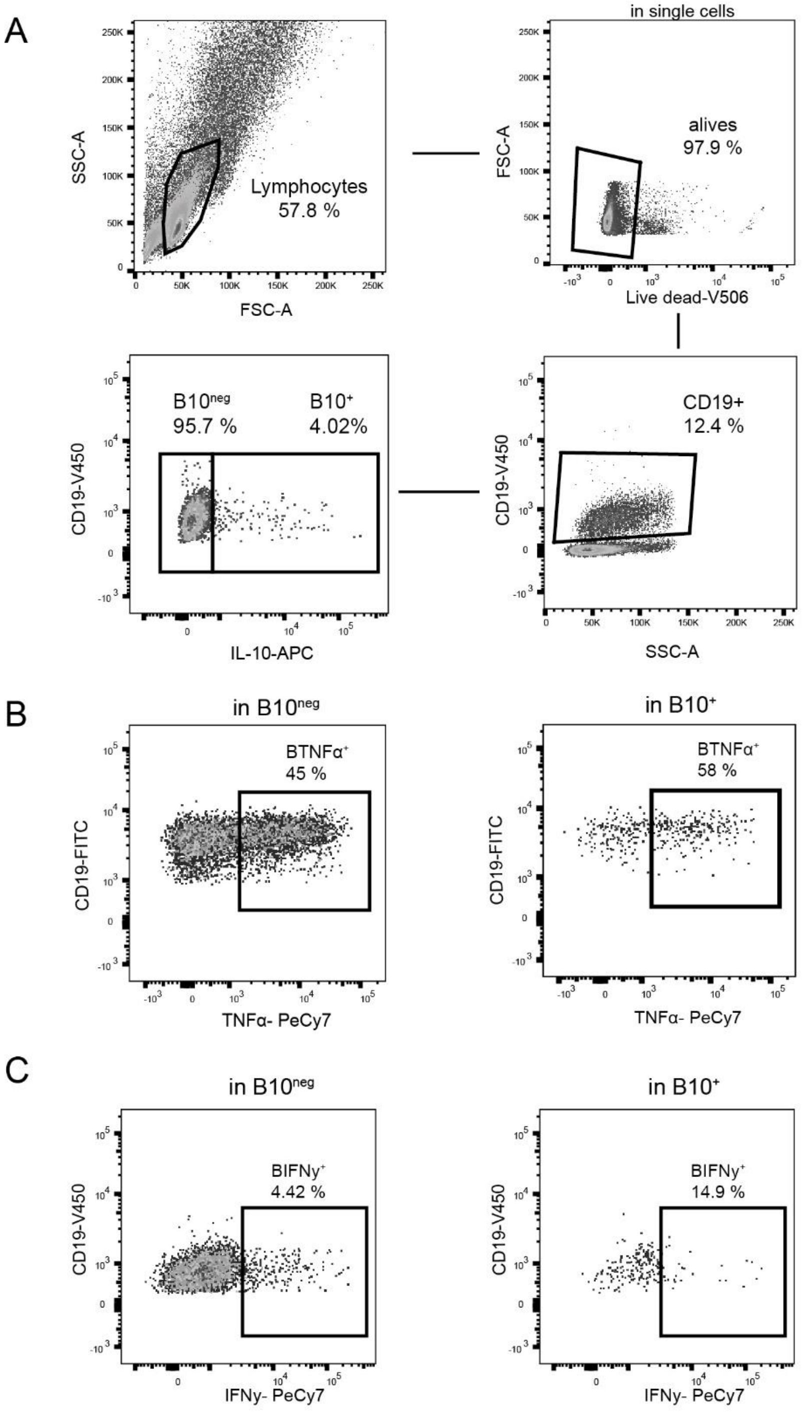

Supplementary Figure 2 : Gating strategy for B10^{+} and B10^{neg} subsets (A). Representative flow cytometry dot plot of BTNFα^{+} **(B)** and BIFNγ^{+} **(C)** frequencies among B10^{+} and B10^{neg} subsets.

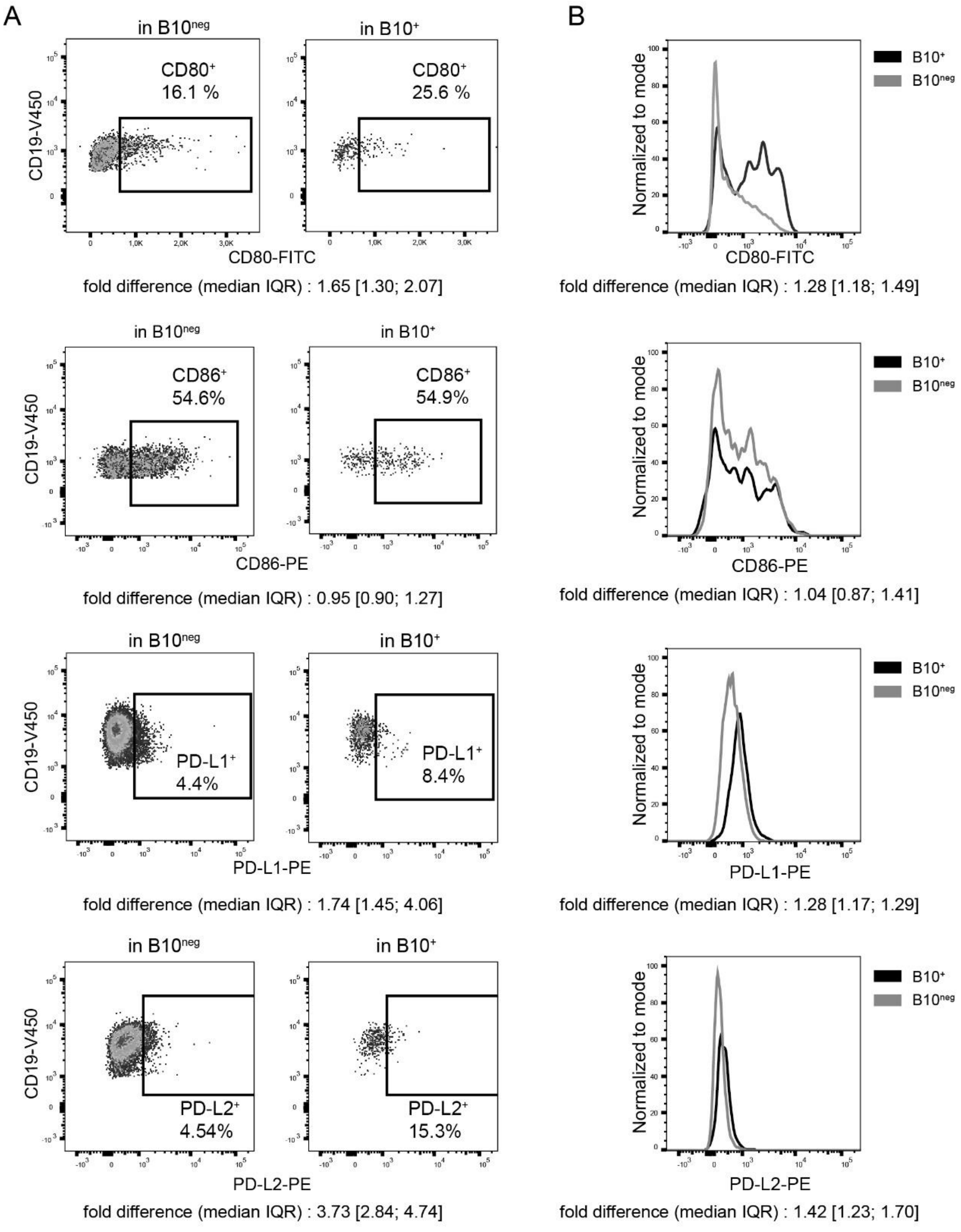

Supplementary Figure 3 : Representative flow cytometry dot plot for CD80, CD86, PD-L1, PD-L2 positive cells among $B10^{neg}$ and $B10^{+}$ subsets (% of $CD19^{+}$) **(A).** Representative flow cytometry histogram overlays of $B10^{neg}$ (light grey) and $B10^{+}$ (black) expression levels of CD80, CD86, PD-L1, PD-L2 **(B)**. Fold difference ($B10^{+}/B10^{neg}$) for expression frequencies and levels are also presented.
